# Transcriptomic data exploration of consensus genes and molecular mechanisms between chronic obstructive pulmonary disease and lung adenocarcinoma

**DOI:** 10.1038/s41598-022-17552-x

**Published:** 2022-08-02

**Authors:** Siyu Zhang, Kun Pang, Xinyu Feng, Yulan Zeng

**Affiliations:** 1 Department of Respiratory Medicine, Liyuan Hospital, Tongji Medical College, Huazhong University of Science and Technology, No. 39 Yanhu Avenue, Wuchang District, Wuhan, 430000 Hubei China; 2 Institute of Genomic Medicine, Wenzhou Medical University, Wenzhou, 325000 Zhejiang China

**Keywords:** Cancer, Drug discovery, Immunology, Biomarkers, Diseases, Oncology

## Abstract

Most current research has focused on chronic obstructive pulmonary disease (COPD) and lung adenocarcinoma (LUAD) alone; however, it is important to understand the complex mechanism of COPD progression to LUAD. This study is the first to explore the unique and jointly molecular mechanisms in the pathogenesis of COPD and LUAD across several datasets based on a variety of analysis methods. We used weighted correlation network analysis to search hub genes in two datasets from public databases: GSE10072 and GSE76925. We explored the unique and jointly molecular mechanistic signatures of the two diseases in pathogenesis through enrichment analysis, immune infiltration analysis, and therapeutic targets analysis. Finally, the results were confirmed using real-time quantitative reverse transcription PCR. Fifteen hub genes were identified: GPI, EZH2, EFNA4, CFB, ENO1, SH3PXD2B, SELL, CORIN, MAD2L1, CENPF, TOP2A, ASPM, IGFBP2, CDKN2A, and ELF3. For the first time, SELL, CORIN, GPI, and EFNA4 were found to play a role in the etiology of COPD and LUAD. The LUAD genes identified were primarily involved in the cell cycle and DNA replication processes; COPD genes we found were related to ubiquitin-mediated proteolysis, ribosome, and T/B-cell receptor signaling pathways. The tumor microenvironment of LUAD pathogenesis was influenced by CD4 + T cells, type 1 regulatory T cells, and T helper 1 cells. T follicular helper cells, natural killer T cells, and B cells all impact the immunological inflammation in COPD. The results of drug targets analysis suggest that cisplatin and tretinoin, as well as bortezomib and metformin may be potential targeted therapy for patients with COPD combined LUAD. These signatures may be provided a new direction for developing early interventions and treatments to improve the prognosis of COPD and LUAD.

## Introduction

Chronic obstructive pulmonary disease (COPD) is a disease characterized by persistent and irreversible airflow limitation. The main symptoms of COPD are coughing sputum and shortness of breath^1,2^. Due to its high morbidity, prevalence, and mortality, COPD has become one of the most impactful chronic respiratory diseases worldwide; it harms human health, resulting in large economic and social burdens. There are many known causes of this disease. Smoking is a risk factor for COPD^3^. Other elements are crucial for the pathogenesis, including immunological reactions, inflammation and heredity risk factors. However, their mechanisms are not fully understood. In conclusion, COPD has negative economic and social consequences. Research is needed on the pathogenesis of COPD. A clear understanding of the risk factors for COPD can promote early intervention, reducing exposure to risk factors and other measures to prevent the occurrence of COPD.

Lung cancer has become one of the most common cancers in the history of the world, according to the latest statistics on all cancers released in 2018^4^. In non-small-cell lung cancer (NSCLC), lung adenocarcinoma (LUAD) is the primary histological subtype of lung cancer, with a high mortality rate and recurrence rate^5^. Although modern therapeutic procedures have made considerable improvements, the recurrent mortality rate remains high. Understanding the clinical significance of proteins, nucleic acids, and other macromolecules in the occurrence and development of LUAD has the potential to help the treatment of disease and related prognosis, and it is also conducive to risk-stratified disease management. To date, most research focuses on how aberrant gene expression and mutations are linked to lung adenocarcinoma progression and carcinogenesis. Consequently, more research into the biology and pathophysiology of LUAD is essential.

It is known that COPD and LUAD exhibit heterogeneity in the clinical presentations and disease progressions. However, the coexistence of COPD and LUAD is common, and progression of COPD to LUAD often has a poor prognosis. Both COPD and LUAD are common respiratory diseases that share similarities. In terms of etiology, COPD and lung adenocarcinoma share common risk factors, such as cigarette smoke exposure, chronic inflammation, gene methylation, and environmental factors^6^. They also have the same clinical symptoms, such as cough and sputum. Persistent bronchial and alveolar inflammatory responses in the long-term course of COPD may have a crucial role in lung cancer induction in the early stages^7^. COPD patients have a higher relative chance of developing lung cancer^8^. In addition, a number of possible mechanisms may explain the relationship between COPD and LUAD, including genetic, epigenetic modifications and oxidative stress factors^9^. Nevertheless, the existing studies have mostly focused on a single disease and the complex disease mechanism between COPD to LUAD is not well understood, and to date no genetic biomarkers have been developed for screening COPD patients who are at high risk for LUAD. Because of the threat to human survival and the burden on social resources, it is extremely important to explore the biological mechanisms to provide a new direction for developing early interventions and treatments to improve the prognosis of COPD and LUAD.

In this study, two large-scale microarray datasets, GSE76925 and GSE10072, were analyzed. Bioinformatics analysis was performed on lung tissue datasets in this work. Although blood is the most commonly available biomarker for histology studies, it is more subject to environmental influences, demographic features, and comorbidities, all of which might cause measurement changes that are undesirable. Lung tissue is more stable than blood, stores more information, and provides more insight into disease mechanisms than blood. Due to the complex evolutionary mechanism of COPD and LUAD, multiple analysis methods were used for multiple datasets, both individually and in combination, to fully analyze the two diseases from multiple perspectives. To gain a deeper understanding of genetic factors, weighted gene coexpression network analysis (WGCNA) in bioinformatics was used to analyze the key genes related to these two diseases, which were both unique and shared, and then combined with Gene Ontology (GO) and Kyoto Encyclopedia of Genes and Genomes (KEGG) to analyze the functions, molecular mechanisms, and pathways of key genes. The immune response in the tumor microenvironment is now an important factor in determining the aggressiveness and progression of tumors. This research aims to assess the immune status of the two diseases by examining the distribution and function of immune cells and investigating connections between the expression of immune cytokines and key genes, which could be crucial for improving COPD and LUAD immunotherapy. Additionally, the Drug-Gene Interaction Database (DGIDB) was used to identify drug targets linked to the disease's pathophysiology, which may guide the early intervention and treatment of patients with COPD and LUAD. Finally, real-time quantitative reverse transcription PCR (RT–qPCR) was used for cytological experimental verification. By exploring the unique and jointly biological mechanisms of the pathogenesis of COPD and LUAD in multiple datasets and from multiple viewpoints, this research will provide new directions for developing early interventions and treatments to improve the prognosis of COPD and LUAD.

## Materials and methods

### Data collection and processing

**Figure 1:**
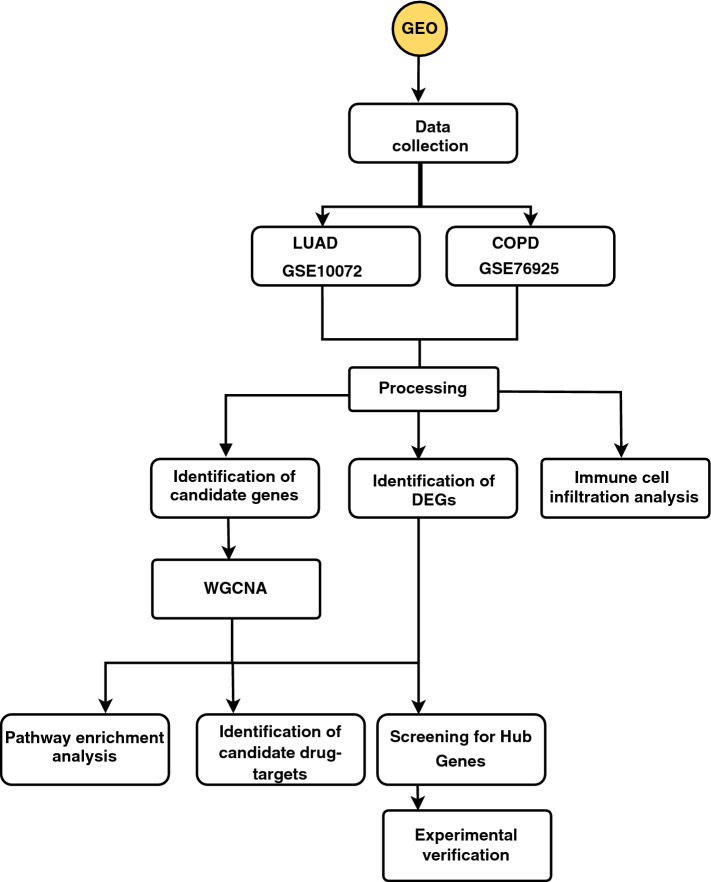
A flowchart showing all the steps involved in our analysis.

We used "COPD," "LUAD," and "Homo sapiens" as keywords to retrieve the transcriptome spectrum of the COPD and LUAD datasets from the Gene Expression Omnibus. We found two expression datasets with readily available data that matched our search criteria, GSE10072 and GSE76925. Gene expression profiles of 58 LUAD patients and 16 healthy subjects (58 lung tumor tissue and 49 normal lung tissue) were found in GSE10072 (Supplementary Table S1). The GSE76925 dataset consisted of gene expression profiles from 111 COPD patients and 40 healthy individuals (Supplementary Table S2). The other detailed messages of the two datasets may be found in Supplementary Table S3. Based on the Robust Multichip Average method of the single-channel Affymetrix chip, we used the Bioconductor Affy package to process and normalize the GSE10072 gene expression data^10^. The data in GSE76925 were processed using the R package to apply a log2 transformation to the original matrix and implement background correction and quantile normalization^11^. We downloaded the two gene expression datasets that had been processed. Subsequently, we matched the probe number with the Gene symbol according to the illuminaHumanv4. DB R package. The probe ID with the highest average expression value was selected when multiple probes were found corresponding to one ID. Then, the Limma package in R was used to identify the differentially expressed genes between COPD and LUAD. Two basic criteria based on the P values and log2FC values of the genes were used to identify differentially expressed genes (DEGs). The corrected P value (adj.P.Value) was obtained. The adj.P.Val < 0.05 and |log2FC|> = 1 were selected as the threshold for DEG screening criteria. Finally, the expression matrices of GSE10072 and GSE76925 were produced. Figure 1 shows a flowchart of all steps involved in our analysis.

### Identification of DEGs and candidate genes

The DEGs between COPD and LUAD were identified using the linear regression model software package in R, Limma. The t test method was used to identify the differentially expressed genes. The *P* value and log2FC value of the genes, as mentioned earlier, were calibrated through multiple experiments, and then the adjustment *P* value (adj. *P*. val) was obtained. The adj. *P*. Val and log2FC used to screen for DEGs. These DEGs were used as a verification set. In the same way, the screening condition adj. *P*. Val < 0.05 was chosen to find candidate genes in the two datasets. We focused on analyses of these genes.

### Weighted gene co-expression network analysis (WGCNA)

Weighted gene coexpression network analysis (WGCNA) is a method of categorizing genes into different modules according to certain conditions to identify biologically relevant information. In COPD-special and LUAD-special sections, we selected candidate genes for WGCNA (adj. *P*. Val < 0.05). To analyze the consensus section, we used the tutorial on the WGCNA website.

To thoroughly explore correlated gene modules, the WGCNA package in R was used to perform WGCNA processing on the candidate genes. First, we clustered the samples in the expression matrix (distinct from the candidate gene clustering described later); the purpose was to assess whether there were obvious outliers in the sample. Datasets containing the corresponding clinical characteristics, and the samples in the datasets were mapped to the clinical characteristics one-by-one. Subsequently, cyberspace construction and module detection were performed. Similar genes were divided into standard modules to obtain a hierarchical clustering dendrogram for module identification. Modules with clinical significance were selected and discussed. Gene significance (GS) and module membership (MM) were two key indicators used to identify modules closely related to clinical features. Finally, we selected modules that were highly related to specific clinical features for further analysis.

### Screening for hub genes

To improve the accuracy of the process of identifying pivotal genes, a method of taking the intersection of the candidate genes after WGCNA analysis and the diseases differential genes were used.

### Pathway enrichment analysis

To perform Gene Ontology (GO) and Kyoto Encyclopedia of Genes and Genomes (KEGG) analyses of genes in significant modules and plot the relevant graphs, we used the “Clusterprofiler” program in R software. Both Go and KEGG data are publicly available databases^12–16^. We also chose adj. *P*. Val < 0.05 as a screening condition in the enrichment analysis.

### Immune cell infiltration analysis

To determine the abundance of 24 types of immune cells in COPD and LUAD, the GSE76925 and GSE10072 gene expression data were uploaded to the Immune Cell Abundance Identifier online analysis website^17^. Box plots were used to graphically represent the difference in immune cell infiltration distribution between the disease group and the normal group. Related heatmaps were produced to visualize the relationship between 24 immune cells and key genes. We used the R packages “ggplot2” and “cowplot” to draw plots. The samples with a *P* value < 0.05 were screened to select meaningful immune cells.

### Identification of candidate drug-targets

To identify the candidate drug targets in COPD and LUAD, the important modules genes from WGCNA analysis were uploaded to the Drug-Gene Interaction Database (DGIDB)^18^. DGIDB was be able to automatically analyze all gene-related drug targets. Finally, the results were plotted using R to map the drug target network plots. The drug with the most nodes was selected as a potential drug target.

### Real-time quantitative reverse transcription PCR

Real-time quantitative reverse transcription PCR (RT–qPCR) was used to verify the expression levels of key genes in COPD and LUAD at the cellular level. The Homo sapiens cell lines of LUAD are H1299 and A549. The COPD cell line was based on the FTC approach and experimental experience, and the normal pulmonary epithelial cell line BEAS-2B was stimulated with 8% CSE smoke for 24 h to form a COPD disease model^19^. All cell lines are derived from ATCC. Total RNA was extracted from cell lines using the TRIzol (Vazyme) reagent. RT–qPCR was performed using the HiScript II Reverse Transcriptase (Vazyme) kit and SYBR Green qPCR MasterMix kit (Seven), and expression levels were normalized to GAPDH and quantified using the 2 − ΔΔ (ct) method. Gene-specific primers synthesized by Gene create company (Wuhan China) are listed in Supplementary Table S4.

## Results

### Identification of DEGs

**Figure 2:**
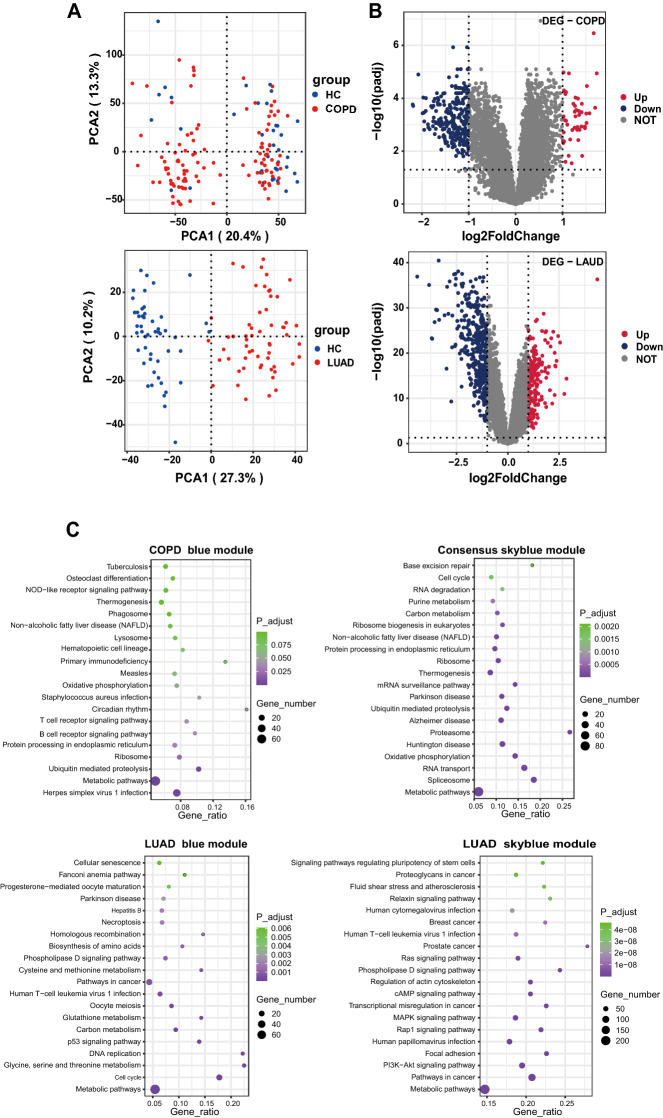
(**A**), the PCA plots showed a clear separation in two datasets. Blue spots represent the control group, and red spots represent the healthy people group. (**B**), volcano graph shows the differential gene expression, upregulated (blue box), downregulated (red box), and unchanged (gray boxes) transcripts. (**C**), KEGG pathway analysis for the target genes of the Important modules. COPD: chronic obstructive pulmonary disease; LUAD: lung adenocarcinoma; KEGG: Kyoto Encyclopedia of Genes and Genomes.

In this study, we used two microarray datasets herein known as GSE10072 and GSE76925. The GSE10072 dataset consisted of 58 lung tumor tissue and 49 normal lung tissue, of which the total number of genes was 12,402. According to the screening conditions adj. *P*. Val < 0.05 and |log2FC|> = 1, we obtained 558 DEG datasets, comprising 185 upregulated and 373 downregulated genes, which were screened out. In addition, 111 experimental data points from COPD patients together with 40 data points from healthy controls were selected from GSE76925. This dataset included 17,130 genes in total. After analyzing, the dataset showed 301 differentially expressed genes; 46 genes were upregulated, and 255 genes were downregulated. The quality of the microarray data was assessed by principal component analysis (PCA). PCA plots showed a clear separation in COPD datasets and LUAD datasets (Fig. 2). Detailed information on all DEGs in LUAD and COPD can be found in Supplementary Table S5.

### Weighted gene co-expression network analysis

#### Disease-specific analysis

**Figure 3:**
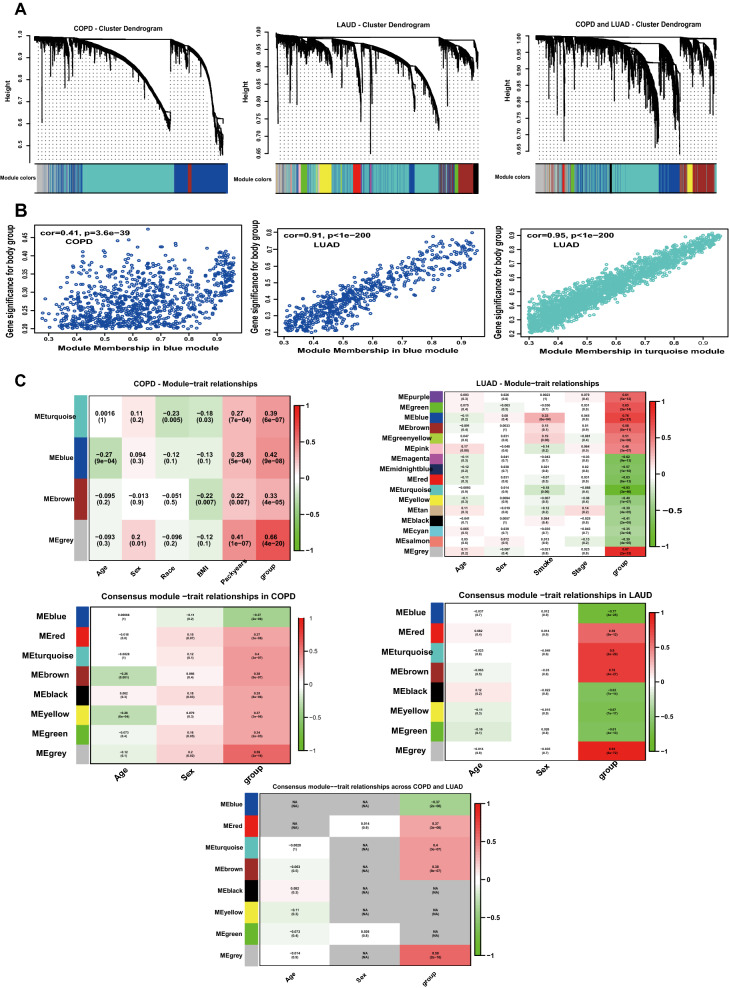
(**A**), the clustering dendrogram of genes. Disease-specific candidate genes with the same functions are denoted by the same color. Gray color denotes genes with unknown function. (**B**), Scatter plot between genes and diseases in important modules. (**C**), Heatmap of module–trait relationships between module genes and clinical traits. In the consensus module, If the two diseases presented similarly in the same clinical trait, the same color was applied to both modules; if the presentation of the diseases was different, then the module was colored gray. COPD: chronic obstructive pulmonary disease; LUAD: lung adenocarcinoma.

Disease-specific candidate genes, 2740 genes in COPD and 4657 in LUAD (adj. *P*. Val < 0.05) were used to establish coexpression network structures unique to each disease. We used the "WGCNA" package in R to construct a scale-free coexpression network. We chose *β* = 8 to maximize the scale-free topology fitting of the candidate gene WGCNA network while maintaining high average connectivity (Supplementary Figure S1). Then, sample clustering was performed to detect outliers in these samples. Outlier values were removed from the set of LUAD samples (Supplementary Figure S1). Supplementary Figure S2 provided clinical features among the candidate genes. Figure 3 showed hierarchical clustering dendrogram of functionally similar genes. We obtained three target modules in COPD and 15 modules in LUAD. One critical module was identified for COPD (denoted in blue) and two were identified for LUAD (denoted in turquoise and blue). The scatter plots of the GS and MM of these modules were also generated (Fig. 3).

#### Consensus network analysis

We chose *β* = 9 to maximize the scale-free topology fitting of the candidate gene WGCNA network (Supplementary Figure S3). If the two diseases presented similarly in the same clinical trait, the same color was applied to both modules; if the presentation of the diseases was different, then the module was colored gray. Thus, a total of 7 modules were obtained for COPD-LUAD. Based on module-trait association and P-value, the turquoise module exhibited the most highly positive correlation (Fig. 3).

### Screening for the hub genes

Candidate genes were identified using the screening conditions of MM and GS. Subsequently, we determined the top 5 to be the essential genes. COPD genes: SH3PXD2B, CORIN, SELL, TRAF3IP3, BHLHE22; LUAD genes: IGFBP2, CDKN2A, MUC5B, CEACAM5, ELF3, MAD2L1, BUB1B, CENPF, TOP2A, ASPM; COPD-LUAD essential genes: GPI, EZH2, EFNA4, CFB, ENO1. The results and screening conditions are reported in Table 1.

**Table 1:** Hub Genes in Target Modules.

Sample	Hub genes	Modules	screening conditions	GS	MM	log2FC	t	P-Value	adj.P.Val
**LUAD**	MAD2L1	Blue	MM > 0.8 and GS > 0.2	0.696	0.953	1.421	10.140	2.11E-17	4.76E-16
	BUB1B	Blue		0.677	0.947	1.421	9.588	3.82E-16	7.09E-15
	CENPF	Blue		0.722	0.941	1.342	10.885	4.19E-19	1.22E-17
	TOP2A	Blue		0.804	0.936	2.456	14.141	2.20E-26	1.73E-24
	ASPM	Blue		0.699	0.935	1.483	10.227	1.33E-17	3.12E-16
**LUAD**	IGFBP2	turquoise	MM from large to small and GS > 0.2	0.418	0.065	1.054	4.901	3.35E-06	1.33E-05
	CDKN2A	turquoise		0.562	0.059	1.020	7.157	1.02E-10	8.56E-10
	MUC5B	turquoise		0.393	0.085	1.211	4.324	3.40E-05	0.000115
	CEACAM5	turquoise		0.603	0.092	2.285	7.970	1.70E-12	1.85E-11
	ELF3	turquoise		0.626	0.094	1.003	8.344	2.50E-13	3.09E-12
**COPD**	SELL	Blue	MM > 0.5 and GS > 0.2	0.304	0.702	1.025	3.934	0.000127	0.001955
	CORIN	Blue		0.342	0.620	1.275	4.481	1.45E-05	0.000485
	SH3PXD2B	Blue		0.402	0.594	1.032	5.398	2.54E-07	5.06E-05
	TRAF3IP3	Blue		0.393	0.584	1.062	5.256	4.91E-07	6.89E-05
	BHLHE22	Blue		0.441	0.546	1.137	6.052	1.07E-08	1.14E-05
**COPD-LUAD**	GPI	turquoise	MM > 0.3 and GS > 0.2	0.317	0.773	0.350	4.079	7.26E-05	0.001336
	EZH2	turquoise		0.267	0.489	0.547	3.407	0.000842	0.006832
	EFNA4	turquoise		0.229	0.630	0.361	2.887	0.004461	0.02228
	CFB	turquoise		0.228	0.484	0.388	2.874	0.004639	0.02287
	ENO1	turquoise		0.209	0.708	0.174	2.590	0.010535	0.041609

Abbreviations: COPD = Chronic obstructive pulmonary; LUAD = lung adenocarcinoma; GS = Gene significance; MM = module membership; log2FC = log2FoldChange; adj.P.Val = adjust P-Value.

### Enrichment analysis

The enrichment analysis included KEGG and GO. KEGG pathway analysis showed that LUAD blue module target genes were significantly enriched in metabolic pathways, the cell cycle, DNA replication, the p53 signaling pathway, pathways in cancer, and necroptosis. GO enrichment analysis showed that target genes concentrated mainly on protein and binding nucleoplasm. KEGG pathway analysis also indicated that turquoise module was predominantly associated with the Rap1 signaling pathway, focal adhesion, MAPK, and PI3K-Akt signaling pathway. GO enrichment analysis showed that genes were mainly in the cytosol and plasma membrane. We conducted KEGG pathway enrichment analysis of the COPD blue module, which identified ubiquitin-mediated proteolysis, ribosome, ubiquitin-mediated proteolysis, and the T/B-cell receptor signaling pathway. GO enrichment results for the blue module included protein binding and cytosol. The KEGG pathways for the consensus turquoise module were oxidative phosphorylation, proteasome, spliceosome, and RNA transport. The GO analysis revealed that the COPD-LUAD turquoise module was primarily enriched in the protein binding nucleoplasm. The detailed results were shown in Fig. 2 and Supplementary Table S6.

### Analysis of the immune cell infiltration

**Figure 4:**
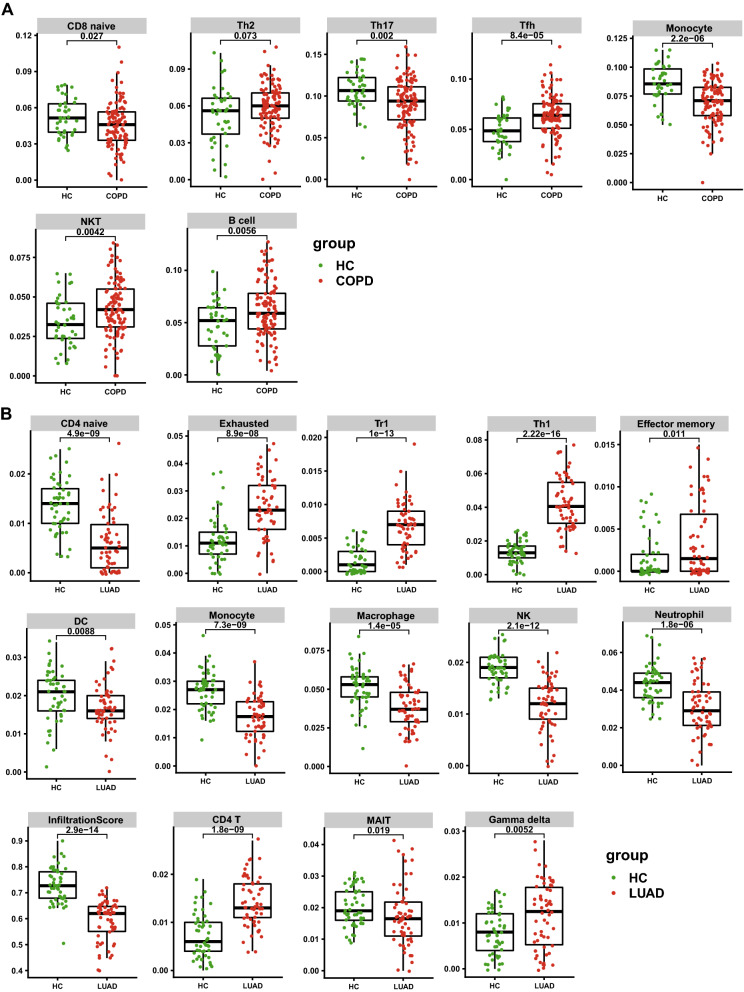
(**A**), the abundance of differentially expressed immune cells in COPD. (**B**), the abundance of differentially expressed immune cells in LUAD. Green represents the control group, and red represents COPD patients. COPD: chronic obstructive pulmonary disease; LUAD: lung adenocarcinoma; HC: control group.

First, we studied the difference in the infiltration of 24 immune cell types in COPD and LUAD patients compared with normal controls. The proportions of follicular helper T cells (Tfh), natural killer T cells (NKT) and B cells in COPD patients from GSE76925 were considerably higher than those in the control group, as shown by the box plots. The proportion of CD8 + naive cells, T helper 17 (Th17) cells, and monocytes were lower in COPD patients than in normal controls. In GSE10072, the abundance of CD4 + T cells, type 1 regulatory T cells (Tr1), and T helper 1 (Th1) cells were higher in LUAD patients than in normal controls. On the other hand, the percentages of CD4 + naive cells, mucosal-associated immune T cells (MAIT), dendritic cells (DC), monocytes, macrophages, natural killer cells (NK), and neutrophils were relatively low. The detailed results were shown in Fig. 4, Supplementary Figure S4 and Supplementary Figure S5. Second, we also made connections between immune infiltrating cells and key genes. NKT cells were strongly positively linked to hub genes, including GPI and ENO1, while Th17 cells were negatively correlated with EZH2 and SELL genes in COPD. The results also revealed that monocytes had a negative correlation with the CFB gene. In addition, the Th1-cell level was most strongly associated with MAD2L1 and GPI in LUAD; in contrast, the CENPF gene was most negatively correlated with macrophages and NK cells (Supplementary Figure S6).

### Identification of candidate drug-targets

**Figure 5:**
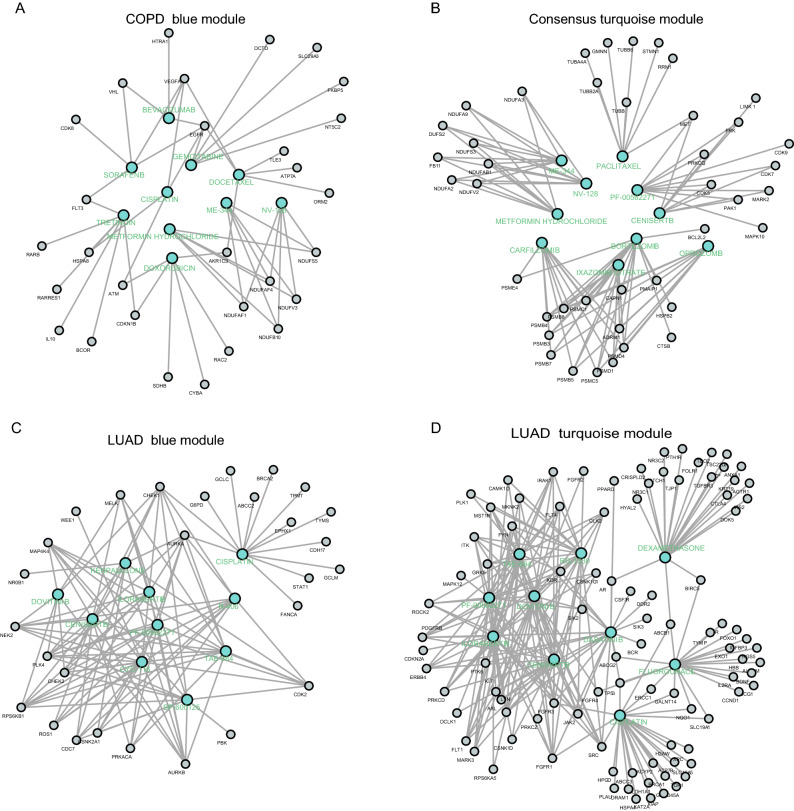
Candidate disease-specific drug-target networks. Label targets and drugs are colored black and turquoise, respectively. The size of the drug letters reveals the degree of the drug. (**A**), Candidate drug-target network of COPD blue module; (**B**), Candidate drug-target network of COPD-LUAD turquoise module; (**C**), Candidate drug-target network of LUAD blue module; (**D**), Candidate drug-target network of the LUAD turquoise module. COPD: chronic obstructive pulmonary disease; LUAD: lung adenocarcinoma.

The drug most frequently found in the LUAD blue module and turquoise module was cisplatin; The prominent drug in the COPD blue module was Tretinoin. In the COPD-LUAD turquoise module, bortezomib and metformin were identified as the main drug- targets. The results obtained from DGIDB were presented in Fig. 5.

### Validation by real-time quantitative reverse transcription PCR

**Figure 6:**
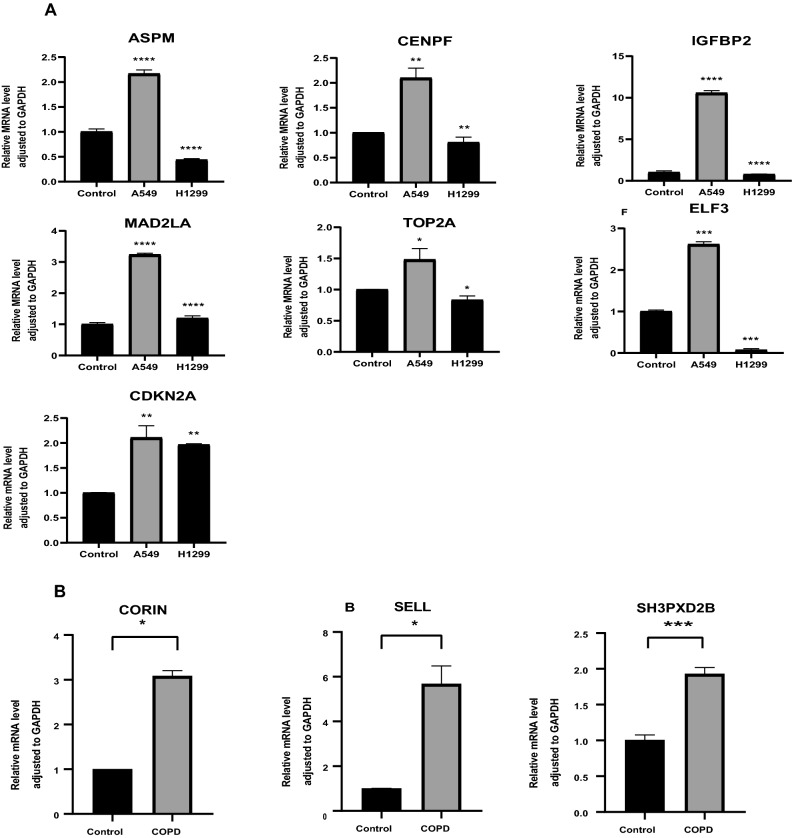
(**A**), RT–qPCR was used to detect the expression of different genes in LUAD and normal cells. (**B**), RT–qPCR was used to detect the expression of different genes in COPD and normal cells. “*” p < 0.05, “**” p < 0.01, “***” p < 0.001. COPD: chronic obstructive pulmonary disease; LUAD: lung adenocarcinoma; RT-qPCR: Real-time quantitative reverse transcription PCR.

RT–qPCR was used to confirm the differential expression of hub genes. Overall, when COPD and LUAD samples were compared to those of normal controls, a total of 10 of the 15 genes analyzed were significantly differentially expressed. The findings were in accordance with the array analysis. COPD-specific genes (CORIN, SELL, SH3PXD2B) and LUAD-specific critical genes (ASPM, CENPF, MAD2L1, TOP2A, CDKN2A, ELF3, IGFBP2) were expressed at higher levels in comparison to normal controls. TRAF3IP3, BHLHE22, MUC5B, CEACAM5, and BUB1B, on the other hand, exhibited no differences in gene expression. The results are shown in Fig. 6.

## Discussion

Because of the threat, they pose to human survival and the toll they take on society, it is extremely important to explore the biological mechanisms of COPD and LUAD, to improve the treatment and prevention of these conditions. COPD and LUAD are both common respiratory diseases, with differences and similarities. Previous studies generally focused on one disease, and little research had been conducted to clarify the complex disease mechanism connecting COPD and LUAD, and to date, no genetic biomarkers have been developed for screening COPD patients who are at high risk for LUAD. To test for differential genes in smoking-related LUAD, Landi et al. created the LUAD-related dataset GSE10072^20^. Morrow et al. generated the COPD-related dataset GSE76925 to analyze global gene transcription^21^. In the current study, the GSE10072 and GSE76925 datasets were analyzed again. However, the enrichment analysis in this study differed from Landi et al. in that it was done after the WGCNA key module was obtained. Unlike Morrow et al. approach the screening of significant genes in this work combined the relevant modules produced by WGCNA with DEGs to improve the accuracy of the results. In addition, immune infiltration study of the diseases and drug targets prediction were carried out, which were not covered in Morrow et al. and Landi et al. investigations. In our present study, we identified the 4 important modules, COPD (blue), LUAD (turquoise and blue) and COPD-LUAD (turquoise), involved in COPD and LUAD pathogenesis by WGCNA using bioinformatics. We screened important key genes in each module and confirmed the findings with RT–qPCR experimental verification. The functions, molecular mechanisms and pathways of key genes were then analyzed in combination with GO and KEGG to provide insight into these genes. We also found significant differences in immune cell infiltration between the disease and normal groups. The roles of key genes involved in many immune responses and immune cell chemotaxis were also found. Meanwhile, we obtained drug targets from the DGIDB database for four modules related to disease pathogenesis. This study bridges the gap between Omics analyses and clinical applications by examining the unique and jointly biological mechanisms of COPD and LUAD pathogenesis in multiple datasets and from multiple perspectives. It may be provided new directions for the development of early interventions and treatments to improve COPD and LUAD prognosis.

We obtained four hub genes, MAD2L1, CENPF, TOP2A, and ASPM, in the LUAD blue module. Mitotic arrest deficiency protein 2 (MAD2L1) and abnormal spindle microtubule assembly (ASPM) have been identified as vital mediators of the chromosomal control pathways. Type IIA topoisomerase (TOP2A) regulates the specific spatial structure (topological structure) by relaxing the positive and negative DNA supercoil structures during DNA replication and transcription and solves the problem of chromosome aggregation and mutual separation of chromatids. The gene located on chromosome 1q41 is centromere protein F, encoding Centromere protein F (CENPF), which is part of the centromere kinetochore complex^22^. The occurrence of most cancers is related to unstable factors in cell formation. Dysregulation of cell cycle pathways, including spindle assembly, resulting in unstable chromosomal structures or massive aneuploidy chromosomal aberrations, leads to tumorigenesis^23^. MAD2L1 participates in motorcycle control of the mitotic spindle assembly checkpoint. Overexpression of MAD2L1 can lead to lung carcinoma susceptibility^24,25^. It was further confirmed in the experiment that the expression of MAD2L1 in LUAD tissue was higher than the average amount. These findings also suggest that increased genetic diversity contributes to altered tumor survival and chemoresistance and that cell cycle pathways, including disruption of spindle assembly, have been the focus of recent chemotherapeutic drug development, such as paclitaxel and colchicine bases.

Through correlation analysis between WGCNA and clinical indicators, we found that the turquoise modules had the strongest negative correlation with disease. The LUAD turquoise module screened for five central genes (IGFBP2, CDKN2A, MUC5B, CEACAM5, ELF3), and these genes in LUAD may be closely related to tumorigenesis. Cyclin-dependent kinase inhibitor 2A (CDKN2A) is an essential cell cycle regulating factor, and a study found that the absence of CDKN2A promoted the progression of lung cancer and that it was correlated with poor survival^26^.

We found that 3 genes were overexpressed in the COPD group (SH3PXD2B, CORIN, SELL). The L-selectin gene (SELL) is also known as CD62 L, which is a type-I transmembrane glycoprotein and cell adhesion molecule. CORIN is a member of the trypsin superfamily of type II transmembrane serine proteases. To date, there is no direct evidence that CORIN and SELL are implicated in the development and progression of COPD. To the best of our knowledge, this is the first time that the association of CORIN and SELL with COPD has been reported. Nevertheless, the effects of CORIN and SELL on inflammation and immunity have been demonstrated^27,28^. SH3 and PX domains 2B (SH3PXD2B) is involved in encoding the cohesive protein of the same name, which triggers the extracellular matrix (ECM) to produce elastase. Furthermore, elastase leads to the degradation of pulmonary elastin, which leads to the occurrence of emphysema, further affecting the formation and progression of COPD ^29^.

The five hub genes in the COPD-LUAD consensus consist of EZH2, EFNA4, CFB, ENO1 and GPI. Interestingly, ENO1 and GPI are the first discoveries of new genes involved in the combined pathogenesis of COPD and LUAD. Enolase 1 (ENO1) glycolytic enzyme catalyzes the transformation of 2-phosphoglycerate to phosphoenolpyruvate to preserve aerobic glycolysis^30^. The present study determined that ENO1 is overexpressed in LUAD, consistent with published studies. ENO1 is involved in proliferative invasion, tumor metastasis and progression in LUAD through glycolysis and the PI3K/Akt pathway^31^. Patients with NSCLC with high ENO1 expression had relatively low disease-free survival and overall survival and were positively correlated with TNM stage^32^. The developmental mechanism of ENO1 and COPD has not been reported. However, it has been reported previously that because COPD is a long-term chronic inflammatory response, the function of neutrophils has a certain degree of influence on its pathogenesis. Neutrophils need to generate intracellular glycogen reserves through the glycolytic pathway to maintain their own cellular functions^33^. The impaired glycolytic pathway involving ENO1 has an impact on neutrophil function. Consequently, these findings support the idea that hat overexpression of these genes plays a significant role in COPD, LUAD, and both, and that they might be therapeutic targets.

The infiltration of immune cells has a crucial function in the progression of illnesses. Finding precise diagnostic markers and assessing the immune cell infiltration pattern in disease has far-reaching implications for improving their prognosis. The lungs of chronic obstructive pulmonary disease patients are prone to inflammation, which is linked to aberrant immunological responses. Immune cell infiltration in COPD and LUAD was considerably different from that in normal controls, according to our findings. The Tfh, NKT and B-cell expression abundance in COPD was considerably higher than that in the control group. Tfh cells are a type of CD4 + T-cell that promotes B-cell survival, affinity maturation, and recombination. However, an overactive Tfh cell response can result in a variety of autoimmune disorders, including rheumatoid arthritis^34^. Tfh cells have been observed in the early stages of COPD (GOLDI/II), which is compatible with the findings of this investigation. B cells and NKT cells are both lymphocytes, which are another type of immune cell^35^. Previous research reported an increase in NKT cells in COPD patients' bronchoalveolar lavage and generated sputum, as well as cytotoxicity against autologous lung epithelial cells^36^. Increased abundance of Tfh, NKT and B cells in patients with chronic obstructive pulmonary disease may help explain the relationship between lung inflammation and immune response in COPD patients, potentially paving the way for disease-targeted immunotherapy.

The richness of CD4 + T cells, Tr1, and Th1 were significantly higher in LUAD samples when compared to that in the control group. CD4 + T cells are essential in host defense, immunological modulation, and autoimmune disease. CD4 + T cells have been found to steadily grow during the shift from normal lung tissue to LUAD^37^. Th1 cells are helper T cells that secrete cytokines that regulate cell development, differentiation, inflammation, and immunological responses^38,39^. Th1 expression was found to be higher in LUAD, which contradicts previous research that found Th1 cytokine levels to be lower in lung cancer patients, Th1 cells to play an antitumor role, and Th2 cells to promote tumor growth^40,41^. The fundamental reason for this is that the research topics are diverse. This research focuses on LUAD, a lung cancer subtype. Second, tumor occurrence and recurrence are complicated processes that may be influenced by patient-specific alterations in Th1 and Th2 cytokines. The exact regulatory mechanism is still a mystery.

In terms of potential drug targets, we identified cisplatin and tretinoin, as well as bortezomib and metformin. Cisplatin chemotherapy is the basis for the treatment of LUAD patients today. Cisplatin chemotherapy function and mechanism of action are related to its cross-linking with guanine and adenine on DNA, obstructing the DNA self-repair mechanism, causing permanent sabotage to DNA, and subsequently inducing carcinoma cell apoptosis^42^. Although there is no reported use of tretinoin for improved prognosis of COPD, a basic study found that Tretinoin significantly treatment abrogated elastase-induced pulmonary emphysema in rats^43^. Bortezomib was consistently identified as an important drug target in the analysis of the COPD-LUAD module. Inhibition of the proteasome leads to disruption of the dynamic balance of proteins, which adversely affects the cellular signaling cascade^44^. Bortezomib and carfilzomib are both proteasome inhibitors and are among the major backbone drugs in oncology therapy. Although bortezomib is primarily used in the treatment regimen for multiple myeloma, it has been reported to have potential benefits in the treatment of lung carcinoma^45^. Recent studies suggest that recognition of the proteasome may be a potential therapeutic target for restoring respiratory muscle function in patients with COPD^46,47^. Metformin may reduce the accumulation of advanced glycation end products by activating amp-related protein kinase (AMPK), thereby reducing airway inflammation, increasing lung capacity, and may improving the prognosis of COPD^48,49^. Furthermore, a previous study reported that use of metformin was inversely associated with pulmonary cancer risk^50^. Consequently, cisplatin and tretinoin, as well as bortezomib and metformin may be potential targeted therapy for patients with COPD combined LUAD. However, there are currently difficulties in repurposing of drug targets. With the exception of cisplatin, which is more likely to cause drug resistance, all of the drug targets we evaluated are currently often used to treat other illnesses. With the gradual development of Druggable Genome, a technique that directly detects genomic sequences to establish the relationship between gene sequence changes and pharmacological effects, we can anticipate drug reuse in the future. It's interesting to note that the technology is currently employed in clinical settings, including genetic testing for cardiovascular medications^51^.

Our research has some limitations. First, the study's samples are limited. More samples and prospective investigations are needed in the next study to fully examine and validate our findings. Second, we lack datasets or lung tissues about COPD combined with LUAD. We will employ clinically matched COPD combined with LUAD samples in the future to confirm the protein expression level of hub genes using western blot analysis. Third, the drug targets identified in this study are based only on a predictive analysis of the disease's major modules and have yet to be empirically validated. In terms of theoretical mechanisms, the medications we acquired may be suitable for improving the prognosis of both disorders. More clinical investigations are needed in the future to establish the validity and reliability of the findings of this study. Moreover, the datasets in this study involved information on demographic characteristics, and differences between disease and control groups regarding demographic characteristics may lead to potential bias in the results of our analysis.

In summary, our research found important genes linked to COPD and LUAD, both individually and jointly. CORIN and SELL, as well as EFNA4 and CFB, were identified for the first time to play a role in the etiology of COPD and LUAD. We discovered the high expression of immune cells in the immunological microenvironment of COPD and LUAD patients. We also found that cisplatin and tretinoin, as well as bortezomib and metformin may be potential targeted therapy for patients with COPD combined LUAD. In fact, we clearly explored the unique and jointly molecular mechanisms of the pathogenesis of COPD and LUAD in multiple datasets and from multiple viewpoints, providing a new direction for developing early interventions and treatments to improve the prognosis of COPD and LUAD.

## Supplementary Information

Supplementary Information 1.

Supplementary Information 2.

## Data Availability

COPD and LUAD datasets from publicly available database: Gene Expression Omnibus. The accession number of both datasets are GSE 10072 and GSE 76925. All the procedures were performed in accordance with the relevant guidelines and regulations. Further inquiries can be directed to the corresponding author.
