# Supplementary material for: Transcriptomic data exploration of consensus genes and molecular mechanisms between chronic obstructive pulmonary disease and lung adenocarcinoma
Source: Sci Rep. 2022 Aug 2;12:13214. doi: 10.1038/s41598-022-17552-x (PMC9345949; doi:10.1038/s41598-022-17552-x)
Supplement: Supplementary file 1 — Supplementary Information 1. [file 41598_2022_17552_MOESM1_ESM.pdf]

Transcriptomic data exploration of consensus genes and molecular mechanisms between chronic obstructive pulmonary disease and lung adenocarcinoma

Authors' names: Siyu Zhang[1], Kun Pang[2], Xinyu Feng[1], Yulan Zeng[1*]

Affiliation: 1. Department of Respiratory Medicine, Liyuan Hospital, Tongji Medical College, Huazhong University of Science and Technology, Wuhan, Hubei, 430000, China

2.Institute of Genomic Medicine, Wenzhou Medical University, Wenzhou, Zhejiang 325000, China

*Corresponding Author: Yulan Zeng

Correspondence: 1989LY0551@hust.edu.cn

Address: Department of Respiratory Medicine, Liyuan Hospital, No. 39 Yanhu Avenue, Wuchang District, Wuhan City 430000, Hubei Province, China

Supplementary Table S1：

Baseline characteristics of the study patients according to the lung adenocarcinoma and normal people

Demographics	LUAD (n=58)	Normal (n=16)	P-value
Age	66.4(± 7.9)	66.6(± 5.7)	0.94
Gender			0.28
Man	35(60.3%)	12(75%)	
Female	23(39.7%)	4(25%)	
Cigarette Smoking Status			0.54
Never	16(27.6%)	4(25%)	
Former	18(31%)	8(50%)	
Current	24(41.4%)	4(25%)	
Stages			0.71
I	22(37.9%)	8(50%)	
II	21(36.2%)	5(31.3%)	
III	12(20.7%)	3(18.8%)	
IV	3(5.2%)	NA	

Abbreviations: LUAD= lung adenocarcinoma.

Supplementary Table S2：

Baseline characteristics of the study patients according to the chronic obstructive pulmonary disease and control group

Demographics	COPD (n=111)	Control (n=40)	P-value
Age (years)	63.3(± 6.6)	65.7(± 9)	0.12
Gender			0.36
Man	52(46.8%)	15(37.5%)	
Female	59(53.2%)	25(62.5%)	
Smoking History (pack-years)	61.3(± 26.3)	33.6(± 21)	P < 0.001
Body Mass Index	25.6(± 4.6)	28.2(± 5.8)	0.02
FEV1 % predicted	26.5(± 9.4)	98.7(± 12.5)	P < 0.001
FEV1/FVC	0.3(± 0.1)	0.8(± 0.1)	P < 0.001
Characteristics			0.91
White	90(81.1%)	34(85%)	
African American	18(16.2%)	5(12.5%)	
Other	3(2.7%)	1(2.5%)	

Abbreviations: COPD= Chronic obstructive pulmonary disease; FEV1=forced expiratory volume in 1 sec; FVC= forced vital capacity.

Supplementary Table S3:

Characteristics and Details of the Microarray Datasets

Datasets	Disease	Grouping		Sample	Platform	Expression Array	Ref.
		Case	Control				
GSE10072	LUAD	58	49	Lung biopsy	GPL96	Affymetrix Human Genome U133A Array	Landi MT et al. 2008
GSE76925	COPD	111	40	Lung biopsy	GPL10558	Illumina HumanHT-12 V4.0 expression beadchip	Morrow JD et a 2017

Supplementary Table S4:

Summarized all primer sequence numbers

Primer	Sequence('to')
ASPM-F	TGATCCACATCTGCTGGAAGGT
ASPM-R	GACAGGATGCAGAAGGATTACT
BHLHE22 -F	GCTGGTTGCCCTTTCTA
BHLHE22 -R	TCACCATCCAACCTGACAAA
BUB1B-F	AAATGACCCTCTGGATGTTTGG
BUB1B-R	GCATAAACGCCCTAATTTAAGCC
CDKN2A-F	TTATTAGAGGGTGGGGTGGATTGT
CDKN2A-R	CAACCCCAAACCACAACCATAA
CEACAM5-F	CGCATACAGTGGTCGAGAGA
CEACAM5-R	TGTAGCTTGCTGTGTCATTT
CENPF-F	TACTGAGTTGAGCCAGAGGGACT
CENPF-R	CATGGTTGTTCTTCGCAGGATAT
CORIN-F	TGCCCAAGCGGAAGTGAG
CORIN-R	GACGGATGGTCCAGGTTGTTT
ELF3-F	ATGGTTTTCGTGACTGCAAGAA
ELF3-R	CAGTACTCTTTGCTCAGCTTTC
GAPDH-F	CTCGCTTCGGCAGCACA
GAPDH-R	AACGCTTCACGAATTTGCGT
IGFBP2-F	GACAATGGCGATGACCACTCA
IGFBP2-R	GCTCCTTCATACCCGACTTGA
MAD2L1-F	GTTCTTCTCATTCGGCATCAACA
MAD2L1-R	GAGTCCGTATTTCTGCACTCG
MUC5B-F	GCCCACATCTCCACCTATGAT
MUC5B-R	GCAGTTCTCGTTGTCCGTCA
TOP2A-F	GCGAGTGTGCTGGTCACTAA
TOP2A-R	ACAATTGGCCGCTAAACTTG

SELL-F	GCCCTCTGTTACACAGCTTCT
SELL-R	GGCCCATAGTACCCCACATC
SH3PXD2B-F	AGATTC TCT TCA GAC GAA
SH3PXD2B-R	GCC TTACAGTATTCATCAA
TRAF3IP3-F	CTCCCATCAAGAAGCCACC
TRAF3IP3-R	TGTAGGGCCTCTTTGAGGTAA

Supplementary Table S6:

GO Biological Process analysis of target modules

Target modules	Term	ID	Input number	P-Value
LUAD Blue module	protein binding	GO:0005515	471	4.28E-123
	nucleoplasm	GO:0005654	213	3
	cytosol	GO:0005829	243	1.61E-59
	nucleus	GO:0005634	228	7.28E-49
	cytoplasm	GO:0005737	204	2.74E-43
	cell division	GO:0051301	61	3.27E-41
	ATP binding	GO:0005524	93	3.18E-29
	DNA replication	GO:0006260	28	1.77E-21
	membrane	GO:0016020	97	4.03E-21
	microtubule binding	GO:0008017	30	1.46E-16
	cytosol	GO:0005829	724	4.66E-125
LUAD turquoise module	plasma membrane	GO:0005886	644	3.25E-105
	cytoplasm	GO:0005737	644	5.05E-105
	nucleus	GO:0005634	632	1.08E-79
	extracellular exosome	GO:0070062	341	2.96E-66
	nucleoplasm	GO:0005654	446	9.53E-55
	integral component of plasma membrane	GO:0005887	237	1.38E-48
	membrane	GO:0016020	283	6.07E-41
	positive regulation of transcription by RNA polymerase II	GO:0045944	192	2.14E-37
	extracellular region	GO:0005576	253	6.56E-37
COPD Blue module	protein binding	GO:0005515	561	6.03E-83
	cytosol	GO:0005829	235	2.19E-24
	nucleus	GO:0005634	236	1.67E-23
	metal ion binding	GO:0046872	134	1.99E-21
	integral component of membrane	GO:0016021	173	9.37E-19
	nucleoplasm	GO:0005654	171	3.32E-18
	cytoplasm	GO:0005737	194	1.14E-15
	mitochondrion	GO:0005739	74	2.51E-12
	regulation of transcription, DNA-templated	GO:0006355	53	2.15E-11
	Golgi apparatus	GO:0005794	62	2.35E-11
COPD-LUAD turquoise	protein binding	GO:0005515	612	4.84E-160
	nucleoplasm	GO:0005654	278	4.39E-85
	cytosol	GO:0005829	321	4.37E-80
	nucleus	GO:0005634	309	1.93E-70
	RNA binding	GO:0003723	153	1.93E-63
	membrane	GO:0016020	164	6.10E-49
	cytoplasm	GO:0005737	241	5.10E-43

	mitochondrion	GO:0005739	97	8.87E-28
	nucleolus	GO:0005730	74	1.64E-24
	extracellular exosome	GO:0070062	121	3.30E-24

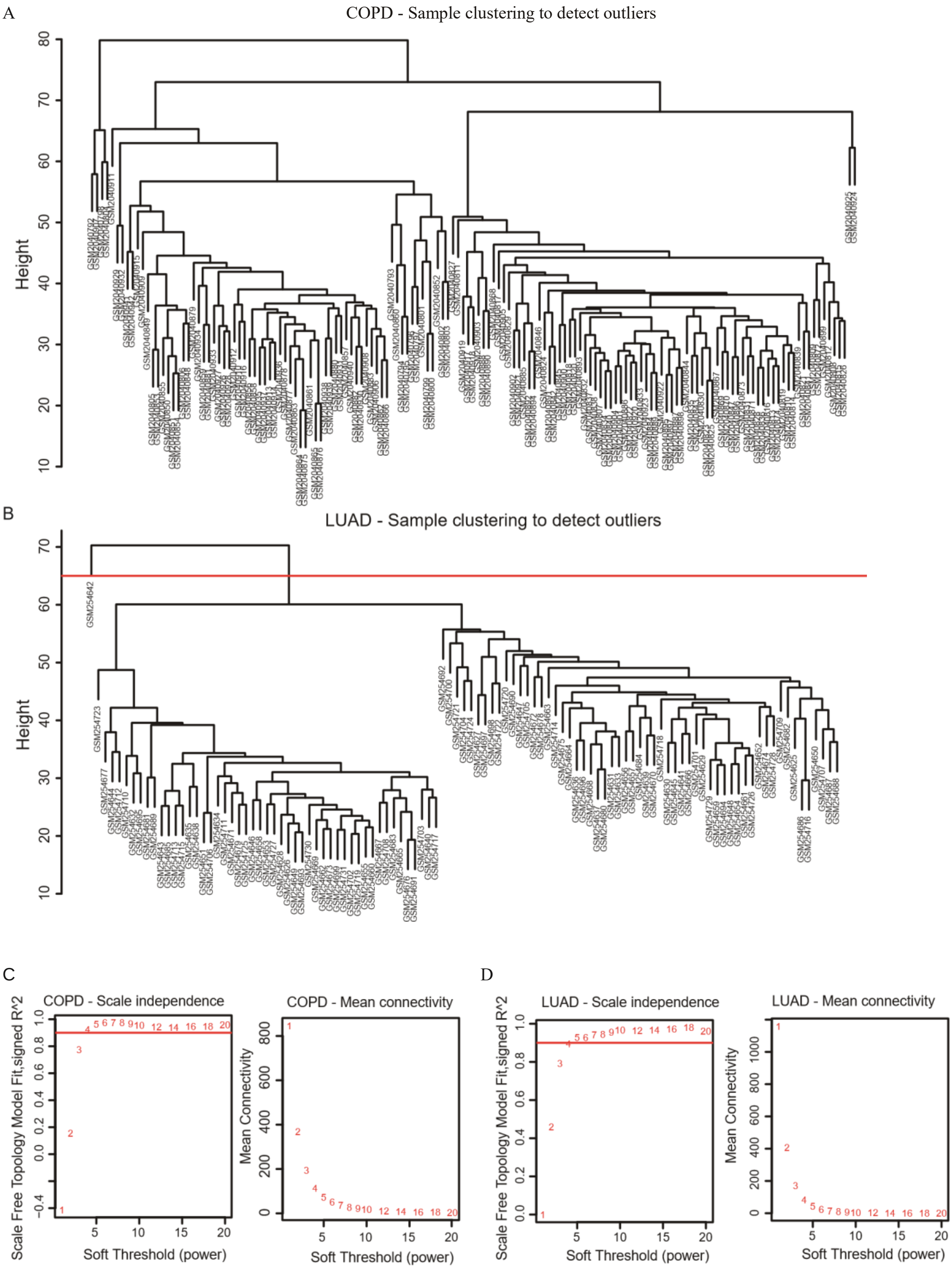

Supplementary Figure S1: Sample clustering to detect outliers in diseases. A, COPD; B, LUAD. We revealed one outlier in LUAD sample; COPD (C) and LUAD (D), $\beta = 8$, which is the lowest power selected to calculate the adjacency of the data. COPD: chronic obstructive pulmonary disease; LUAD: lung adenocarcinoma.

A

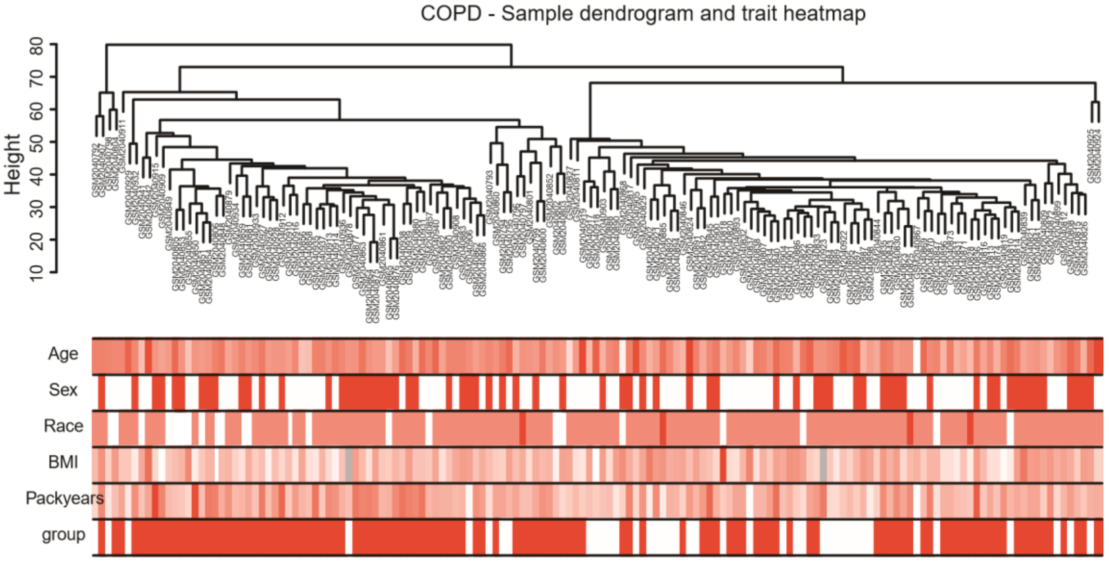

B

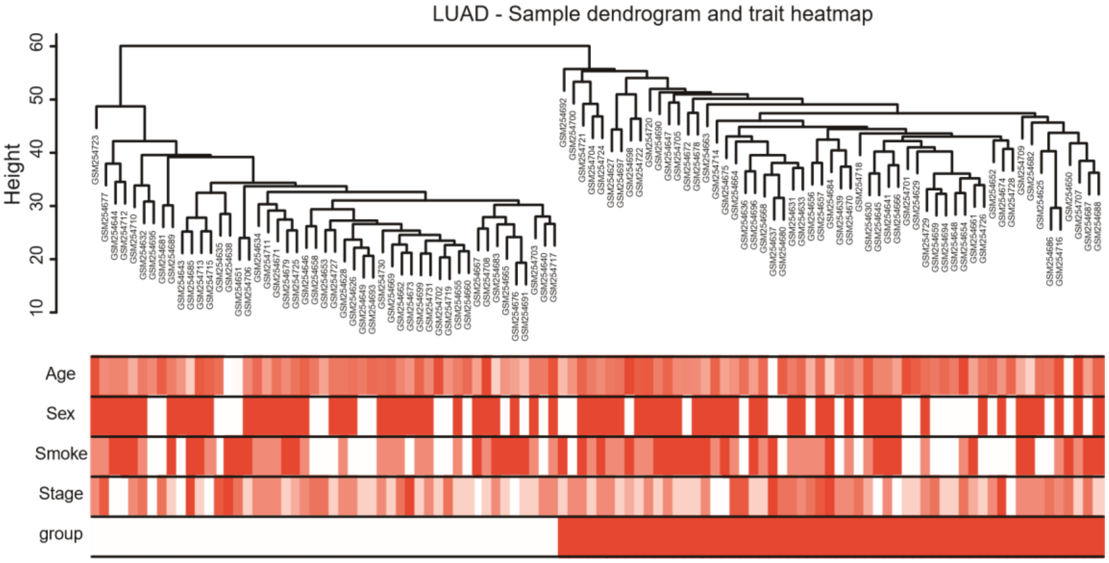

Supplementary Figure S2: Sample dendrogram and trait heatmap in diseases. A, COPD; B, LUAD. In the heatmap, the ordinate represents the clinical information "Age," "Sex," "Race," "BMI," "Pack-Years," "Group," and the abscissa represents a color scale from red to white, where red denotes high expression, white denotes low expression, and gray denotes lack of input. COPD: chronic obstructive pulmonary disease; LUAD: lung adenocarcinoma.

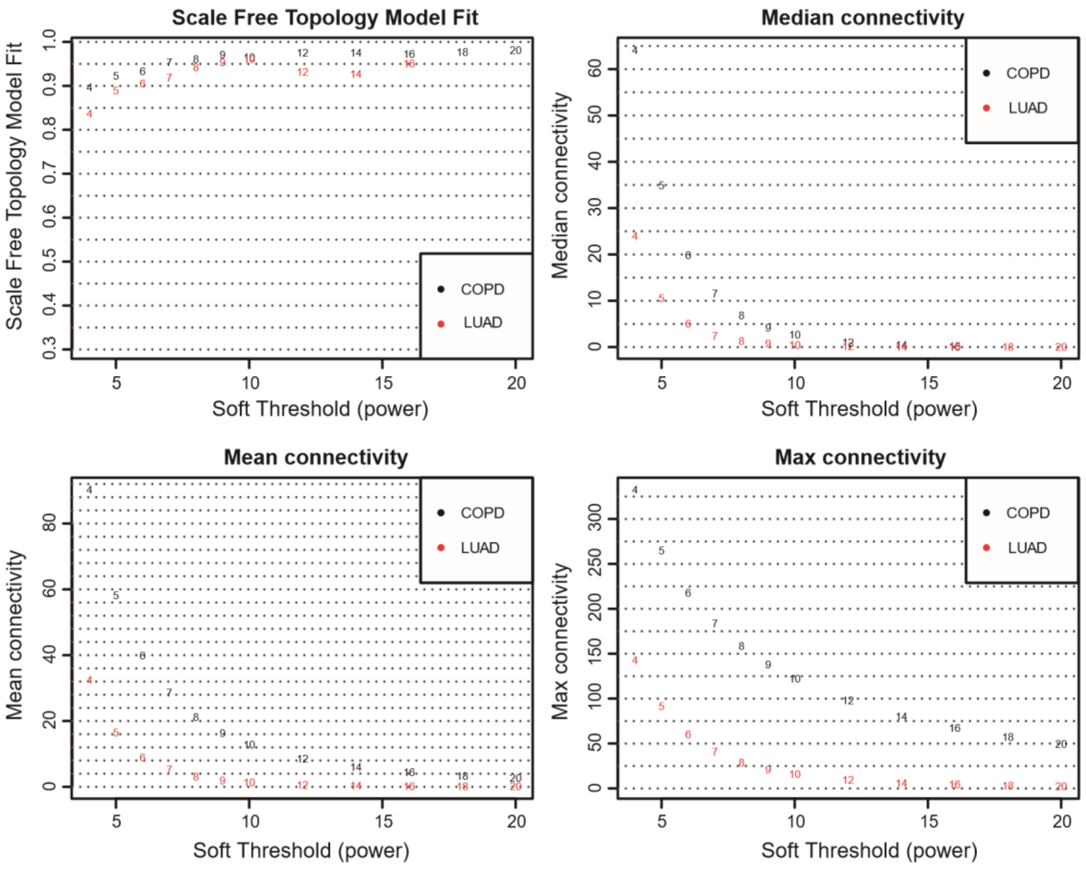

Supplementary Figure S3: β= 9, which is the lowest power was selected to calculate the adjacency of the data.

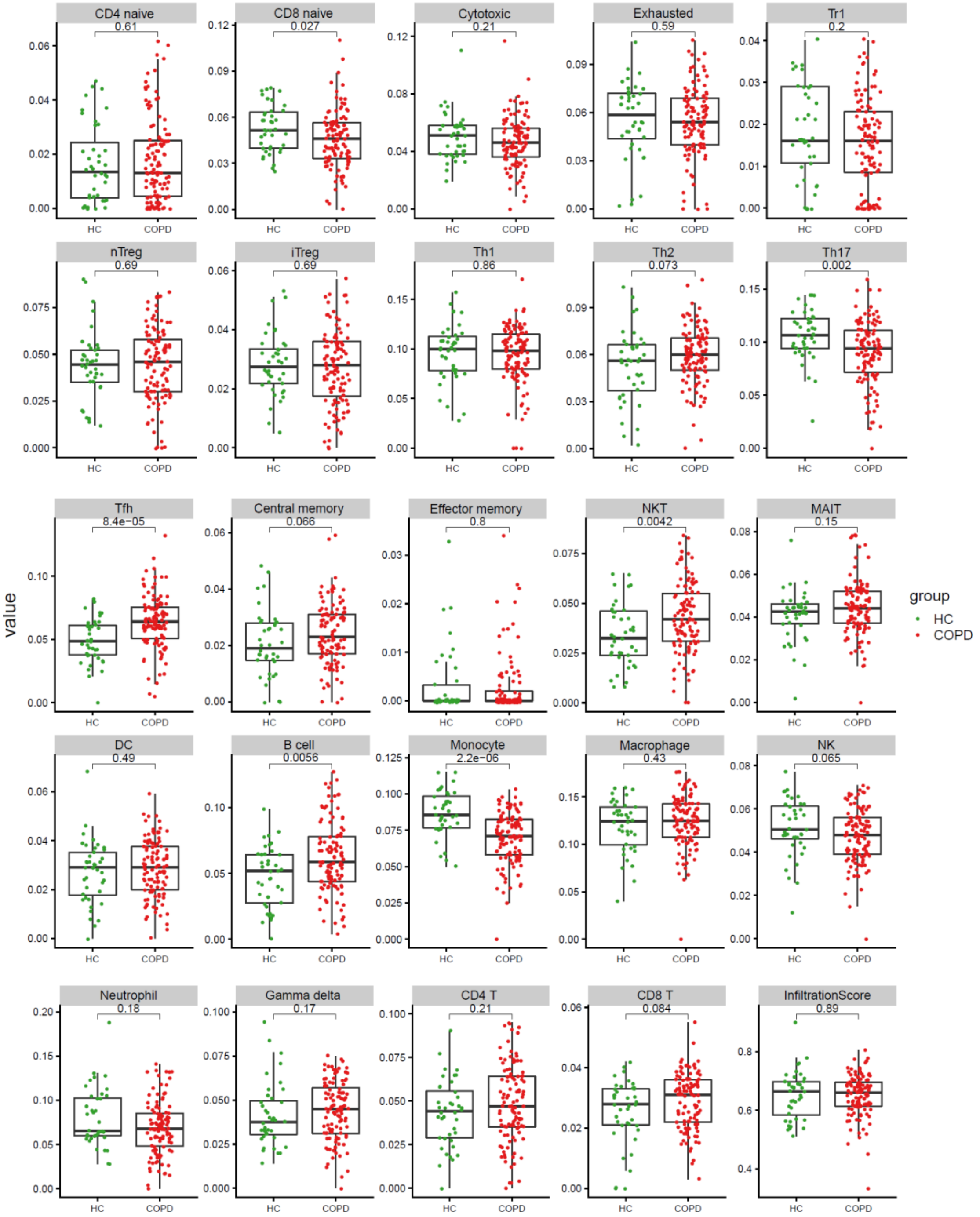

Supplementary Figure S4: The abundance of 24 types of immune cells in COPD. Green represents the control group, and red represents COPD patients. COPD: chronic obstructive pulmonary disease.

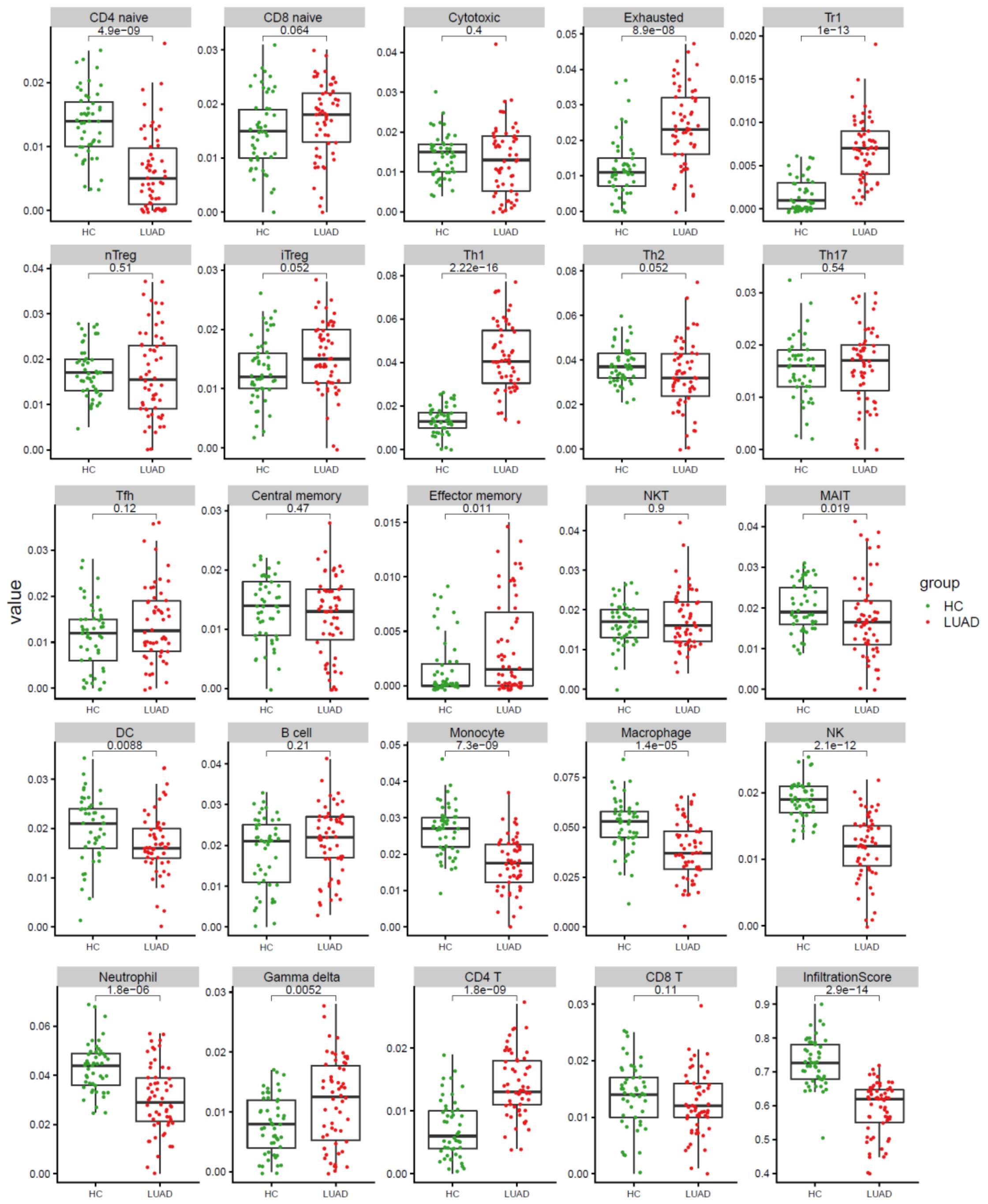

Supplementary Figure S5: The abundance of 24 types of immune cells in LUAD. Green represents the control group, and red represents LUAD patients. LUAD: lung adenocarcinoma.

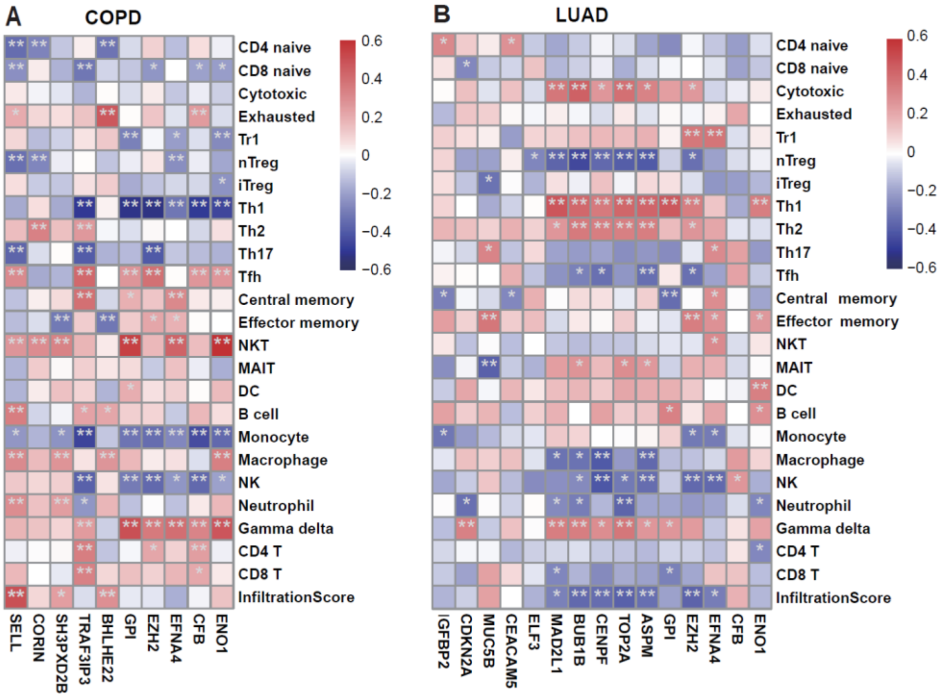

Supplementary Figure S6: Relationship of 24 immune cells with COPD, LUAD and COPD-LUAD central genes. Red represents positive correlations, blue represents negative correlations, and white represents no significance. The darker the color is, the greater the correlation. A plot indicates COPD. The B plot indicates LUAD. "*"$p < 0.05$, "**"$p < 0.01$. COPD: chronic obstructive pulmonary disease; LUAD: lung adenocarcinoma.
